# Screen Time from Adolescence to Adulthood and Cardiometabolic Disease: a Prospective Cohort Study

**DOI:** 10.1007/s11606-022-07984-6

**Published:** 2023-01-10

**Authors:** Jason M. Nagata, Christopher M. Lee, Feng Lin, Kyle T. Ganson, Kelley Pettee Gabriel, Alexander Testa, Dylan B. Jackson, Erin E. Dooley, Holly C. Gooding, Eric Vittinghoff

**Affiliations:** 1 Department of Pediatrics, University of California, San Francisco, 550 16th Street, 4th Floor, Box 0503, San Francisco, CA 94143 USA; 2 Department of Epidemiology and Biostatistics, University of California, San Francisco, San Francisco, USA; 3 Factor-Inwentash Faculty of Social Work, University of Toronto, Toronto, ON Canada; 4 Department of Epidemiology, University of Alabama at Birmingham, Birmingham, AL USA; 5 Department of Management, Policy and Community Health, University of Texas Health Science Center at Houston, Houston, TX USA; 6 Department of Population, Family, and Reproductive Health, Johns Hopkins Bloomberg School of Public Health, Johns Hopkins University, Baltimore, MD USA; 7 Division of General Pediatrics and Adolescent Medicine, Department of Pediatrics, Emory University School of Medicine, Atlanta, GA USA; 8 Children’s Healthcare of Atlanta, Atlanta, GA USA

**Keywords:** Screen time, Adolescents, Television, Videos, Technology, Diabetes, Hypertension, Obesity, Hyperlipidemia

## Abstract

**Background:**

Previous studies have analyzed the relationship between screen time and cardiometabolic disease risk factors among adolescents, but few have examined the longitudinal effects of screen time on cardiometabolic health into adulthood using nationally representative data.

**Objective:**

To determine prospective associations between screen time and later cardiometabolic disease over a 24-year period using a nationally representative adolescent cohort.

**Design:**

Longitudinal prospective cohort data from the National Longitudinal Study of Adolescent to Adult Health (Add Health) collected from 1994 to 2018.

**Participants:**

Adolescents aged 11–18 years old at baseline (1994–1995) followed for 24 years.

**Main Measures:**

Predictors: screen time (five repeated measures of self-reported television and video watching from adolescence to adulthood). Outcomes: Five repeated measures of body mass index (BMI); two repeated measures of waist circumference, hypertension, hyperlipidemia, and diabetes collected at 15- and 24-year follow-up exams.

**Key Results:**

For the 7105 adolescents in the sample (49.7% female, 35.0% non-white), the baseline adolescent average screen time per day was 2.86 ± 0.08 hours per day, which generally declined through 24-year follow-up. Average BMI at baseline was 22.57 ± 0.13 kg/m^2^, which increased to 30.27 ± 0.18 kg/m^2^ through follow-up. By 24-year follow-up, 43.4% of participants had obesity, 8.4% had diabetes, 31.8% had hypertension, and 14.9% had hyperlipidemia. In mixed-effects generalized linear models, each additional hour of screen time per day was associated with 0.06 (95% CI 0.04–0.09) within-person increase in BMI. Each additional hour of screen time per day was associated with higher within-person odds of high waist circumference (AOR 1.17, 95% CI 1.09–1.26), obesity (AOR 1.09, 95% CI 1.03–1.15), and diabetes (AOR 1.17, 95% CI 1.07–1.28). Screen time was not significantly associated with hypertension or hyperlipidemia.

**Conclusions:**

In this prospective cohort study, higher screen time in adolescence was associated with higher odds of select indicators of cardiometabolic disease in adulthood.

**Supplementary Information:**

The online version contains supplementary material available at 10.1007/s11606-022-07984-6.

## INTRODUCTION

Children, adolescents, and adults are spending an increasing amount of time engaging in sedentary behaviors.^1,2^ A growing body of evidence suggests that sedentary behaviors, including screen-related activities such as watching television and videos, may increase the risk of cardiometabolic disease.^1–3^ Mechanisms to explain the association between screen time and cardiometabolic disease include the promotion of unhealthy behaviors such as excess caloric intake and displacement of physical activity.^4^ According to the Bureau of Labor Statistics, from 2013 to 2017, 80% of the United States (U.S.) population ages 15 and older watched television on a given day, which accounted for 55% of all time spent in leisure and sports.^5^ Although contemporary screen modalities have expanded to smartphones and other devices, television and video watching remain the most commonly used screen modalities in adolescents and adults.^5–8^

Previous studies have examined the cross-sectional associations of screen time and cardiometabolic risk factors, including systolic blood pressure, non-HDL cholesterol, glucose, and waist circumference in young children, adolescents, and college-aged adults. In particular, screen time has been associated with higher risk of type 2 diabetes in both children and adults, higher non-HDL cholesterol in young children, and obesity in both children and adolescents.^9–12^ However, as most of these existing studies have cross-sectional designs, few have analyzed the longitudinal associations between screen time and cardiometabolic health outcomes with nationally representative data. Longitudinal studies are essential to better understand the impact of screen time on adult health risks over time.

Prior studies conducted in the U.S. have also shown that adolescent screen use is prospectively associated with binge-eating disorder^13^ and higher body mass index (BMI)^14^ at 1-year follow-up using a national cohort study of children (9–10 years old). To our knowledge, there has been a paucity of longitudinal studies exploring the association between child and adolescent screen time and adult health over a period of 20 years or more. One exception is a 2004 New Zealand study that followed a birth cohort to age 26, and found that television viewing was associated with increased weight and cholesterol levels in adulthood.^15^ While previous research offers some insight into the long-lasting adverse effects of screen time on health, the current study extends this knowledge by examining the findings in an older age cohort when cardiometabolic disease may be more prevalent (e.g., heart disease is the second leading cause of death between ages 35 and 44)^16^ and in a larger, nationally representative sample in the U.S.

To address the gaps in the literature, the current study aims to determine prospective associations between screen time and cardiometabolic disease using a U.S. national longitudinal cohort of adolescents followed over a 24-year period. We hypothesize that higher screen time in adolescence through adulthood is associated with greater cardiometabolic disease in adulthood. The findings can inform the development of guidelines for adolescent screen time and sedentary behaviors as a preventive measure for cardiometabolic disease in later life.

## METHODS

### Study Population

This study utilizes data from the National Longitudinal Study of Adolescent to Adult Health (Add Health), a longitudinal cohort study of a nationally representative sample of adolescents starting in grades 7–12 in the U.S. who have been followed into adulthood. Data were collected from five waves of interviews: Wave I (1994–1995; age 11–18) to Wave V (2016–2018; age 33–43). The initial wave included 20,745 adolescents in grades 7–12 sampling from 132 schools throughout the U.S., representative for race/ethnicity, religion, size, and urban versus rural environments during the 1994–1995 school year. Attrition by wave is shown in ESM Appendix A. Overall, 7295 participants were followed across all five waves. Of those, 7105 had valid screen time exposure and at least one cardiometabolic disease outcome data across all five waves and were included in this analysis. Additional details of the study design have been reported elsewhere.^17^ The University of North Carolina Institutional Review Board approved the Add Health data collection and the University of California, San Francisco Institutional Review Board approved this specific secondary analysis.

### Data Collection

Individuals recruited from the school environment were invited to join the longitudinal cohort and participate in an in-home interview. An interviewer then traveled to the home or another suitable location of the participant in Waves I through IV for an in-home interview. In Wave V, there was a mixed-mode survey design (i.e., combination of web or mail surveys with a minority receiving in-person interviews) which is described in detail elsewhere.^18^ All waves collected information about screen use. Height and weight were measured by the interviewer at the end of the interview in Waves II through IV, and at an in-person visit after completion of the survey in Wave V. Waist circumference, cardiovascular measures including blood pressure, and metabolic measures including glycosylated hemoglobin (hemoglobin A1c) and cholesterol levels (HDL-C and LDL-C), were additionally collected at the end of the in-home interview in Wave IV and at an in-person visit after completion of the survey in Wave V.^19–21^

### Measures

#### Exposure: Screen Time

In all waves, participants responded to the following questions: “How many hours a week do you watch television?” and “How many hours a week do you watch videos?” The screen time measure summed the hours per week for television and videos, and converted these into daily estimates (dividing the hours per week by seven).^22–25^ Self-reported measures of television viewing have demonstrated a significant moderate positive correlation (Spearman’s *p* = 0.54, *p* < 0.001) with objective measures (e.g., electronic television monitor) and a high level of agreement, with 95% of values within 4 hours of the mean.^26^ Self-reported hours per week of television watching has demonstrated acceptable test-retest reliability (7-day test-retest intraclass correlations 0.76–0.81).^27,28^

#### Outcomes

The primary outcome measures of this study are listed below and included measurements of several cardiometabolic disease risk factors, such as BMI at all waves, and waist circumference, diabetes, hypertension, and hyperlipidemia at Waves IV and V.

#### BMI

BMI was computed using the standard formula weight (kilograms) divided by height (meters) squared (BMI = weight/height^2^). Weight (Health-o-meter 844KL High Capacity Digital Bathroom Scale; Jarden Corporation; Rye, NY) and height (Carpenter’s square, steel tape measure) were assessed by the interviewer with the participant in light clothes and without shoes in Waves II through V, and by self-report in Wave I and for half of Wave V without measured data.

#### Obesity

BMI was converted into sex- and age-specific percentiles in accordance with the Centers for Disease Control and Prevention (CDC) growth curves and definitions. Individuals under 18 years old were classified as having obesity if their BMI was ≥ 95^th^ percentile.^29^ Individuals 18 and older were classified as having obesity if their BMI was ≥ 30 kg/m^2^.^30^

#### Waist Circumference

Waist circumference was classified according to the National Institutes of Health Clinical Guidelines on the Identification, Evaluation, and Treatment of Overweight and Obesity in Adults using sex-specific thresholds identifying increased relative risk for the development of obesity-associated risk factors. High waist circumference was defined as > 102 cm for males and > 88 cm for females.^31^

#### Diabetes

Respondents in Waves IV and V were determined to have diabetes if they had levels of fasting glucose ≥ 126 mg/dL, non-fasting glucose ≥ 200 mg/dL, hemoglobin A1c ≥ 6.5%,^32^ a self-reported history of diabetes (except during pregnancy), or anti-diabetic medication use in the 4 weeks preceding the assessment.

#### Hypertension

Blood pressure was calculated using the mean of two measurements separated by a 30-s interval from a factory-calibrated, Microlife BP3MC1-PC-IB oscillometric blood pressure monitor (MicroLife USA, Inc.; Dunedin, FL). Hypertension was classified as a measured systolic blood pressure ≥ 130 mmHg or a measured diastolic blood pressure ≥ 80 mmHg based on the 2017 American College of Cardiology/American Heart Association Task Force on Clinical Practice Guidelines,^33^ anti-hypertensive medication use in the 4 weeks preceding the assessment, or an affirmative response to the query: “Have you ever been diagnosed with high blood pressure or hypertension?”

#### Cholesterol

Hyperlipidemia was defined as total cholesterol corresponding to ≥ 240 mg/dL in the nationally representative National Health and Nutrition Examination Surveys,^34,35^ antihyperlipidemic medication use in the 4 weeks preceding the assessment, or an affirmative response to the question: “Have you ever been diagnosed with high cholesterol, triglycerides, or lipids?”

#### Additional Covariates

Additional covariates included self-reported demographics such as age, sex, race/ethnicity, parents’ highest education (high school or less versus college or more), and household income (percent of the federal poverty line), as these have been shown to be associated with both screen time ^36^ and cardiometabolic disease risk.^37^ Baseline data from parents’ self-report of household income in the last calendar year was used to evaluate household income as a covariate. Gaussian normal regression imputation models were used to impute the income for the 1638 parents who did not respond, stated that they did not know, or were uncomfortable answering the income question, similar to the method used in previous studies.^38^ The household income variable was then transformed into a continuous measure that was a ratio of household income relative to the federal poverty level. Behavioral covariates that are associated with screen time use and metabolic disease^34,39–41^ were assessed at each wave, including self-reported alcohol use in the past 12 months (yes/no) and lifetime smoking (yes/no).

### Statistical Analysis

Data analyses were performed using Stata 17.1 (College Station, TX). We averaged daily screen time, our primary exposure, across multiple waves from baseline through the wave where change since baseline in the repeated outcomes was assessed. Specifically, the average of screen time in Waves I and II was used to predict change in the outcomes from baseline to Wave II, and screen time averaged over Waves I–V for changes from baseline to Wave V. For continuous outcomes, we used a multilevel mixed-effects generalized linear model with random effects for participant to estimate the association of average screen times and changes since baseline in study outcomes, first unadjusted, then adjusting for age (time varying), sex, race/ethnicity, smoking (time varying), alcohol (time varying), parent education (Wave I), income to poverty ratio (Wave I), and BMI (Wave I). Covariates in the adjusted model were specified a priori, based on our reading of the literature.^34,39–41^ We used analogous unadjusted and adjusted pooled logistic models with robust standard errors clustered by participant to estimate the associations of daily screen time with incidence of binary cardiometabolic disease risk factors. The binary obesity outcome included five repeated measures from Wave I to Wave V. The binary high waist circumference, diabetes, hypertension, and hyperlipidemia outcomes included two repeated measures from Wave IV to Wave V. Goodness of fit was assessed by comparisons with larger models including quadratic and interaction terms. All analyses incorporate Add Health’s preconstructed sample weights to provide nationally representative estimates.^42^

### Sensitivity Analyses

We ran several sensitivity analyses for the models. First, we ran the above analyses excluding participants who were obese at baseline (ESM Appendix A). Second, we examined objective (e.g., laboratory values or blood pressure measurements and/or medication history) outcomes separately from self-reported outcomes (ESM Appendix B). Third, given lack of blood pressure and hemoglobin A1c data prior to Wave IV, we examined the association between screen time (averaged across Wave IV and V) and systolic and diastolic blood pressure, and hemoglobin A1c, adjusting for the respective measurement at Wave IV.

## RESULTS

For the 7105 adolescents in the sample (Table 1, 49.7% female, 35% non-white), the baseline average screen time per day was 2.86 ± 0.08 h, which generally declined through adulthood (ages 33–43). Average BMI at baseline was 22.57 ± 0.13 kg/m^2^, which increased to 30.27 ± 0.18 kg/m^2^ through adulthood. By adulthood, 43.4% of participants had obesity, 8.4% had diabetes, 31.8% had hypertension, and 14.9% had hyperlipidemia.

**Table 1:** Descriptive and Health Characteristics of 7105 Participants in the National Longitudinal Study of Adolescent Health

	Total	Screen time quartiles (Wave I)			
		Quartile 1	Quartile 2	Quartile 3	Quartile 4
	Mean ± SE / %^a^	Mean ± SE / %^a^	Mean ± SE / %^a^	Mean ± SE / %^a^	Mean ± SE / %^a^
**Demographic characteristics**					
Sex					
Male	50.3%	42.2%	51.6%	53.2%	54.7%
Female	49.7%	57.8%	48.4%	46.8%	45.3%
Race/ethnicity					
White (non-Hispanic)	65.0%	67.5%	69.7%	68.0%	55.0%
Black/African American (non-Hispanic)	15.9%	11.8%	11.8%	13.5%	26.1%
Hispanic/Latino	12.2%	12.9%	11.6%	11.0%	13.3%
Asian/Pacific Islander (non-Hispanic)	3.8%	4.3%	3.3%	5.0%	2.6%
American Indian/Native American	2.4%	2.7%	3.2%	1.6%	2.4%
Other	0.7%	0.9%	0.5%	0.8%	0.6%
Parents with college education or more	65.7%	65.8%	67.6%	69.5%	59.4%
Percent of federal poverty level^b^	1.47 ± 0.05	1.59 ± 0.08	1.57 ± 0.07	1.48 ± 0.06	1.26 ± 0.04
**Health behaviors**					
Smoking ever (Wave I)	59.1%	56.5%	58.1%	57.9%	64.1%
Alcohol ever (Wave I)	55.7%	53.0%	56.0%	55.8%	58.1%
**Screen time (hours per day, mean ± SE, median (IQR))**					
Wave I (ages 11–18)	2.86 ± 0.08, 2.14 (1.00–3.86)	--	--	--	--
Wave II (ages 11–18)	2.65 ± 0.07, 2.21 (1.21–3.64)	--	--	--	--
Wave III (ages 18–26)	2.68 ± 0.07, 2.29 (1.43–3.52)	--	--	--	--
Wave IV (ages 24–32)	1.85 ± 0.03, 2.14 (1.36–3.21)	--	--	--	--
Wave V (ages 32–42)	1.95 ± 0.05, 2.09 (1.37–3.03)	--	--	--	--
**Cardiometabolic outcomes, continuous**					
Body mass index (kg/m^2^**)**					
Wave I (ages 11–18)	22.57 ± 0.13	22.27 ± 0.15	22.50 ± 0.19	22.42 ± 0.22	23.09 ± 0.22
Wave II (ages 11–18)	23.27 ± 0.14	22.83 ± 0.17	23.17 ± 0.21	23.16 ± 0.22	23.94 ± 0.23
Wave III (ages 18–26)	26.62 ± 0.15	26.18 ± 0.23	26.26 ± 0.22	26.55 ± 0.24	27.52 ± 0.30
Wave IV (ages 24–32)	29.22 ± 0.18	28.57 ± 0.28	28.87 ± 0.27	29.00 ± 0.26	30.45 ± 0.33
Wave V (ages 32–42)	30.27 ± 0.18	29.53 ± 0.25	29.99 ± 0.25	30.19 ± 0.26	31.39 ± 0.30
Waist circumference (cm)					
Wave IV (ages 24–32)	98.61 ± 0.43	96.53 ± 0.62	98.26 ± 0.56	98.10 ± 0.59	101.62 ± 0.76
Wave V (ages 33–43)	98.75 ± 0.61	96.17 ± 0.98	97.98 ± 1.03	98.00 ± 0.82	102.87 ± 1.05
**Cardiometabolic disease outcomes, binary**					
Obesity					
Wave I (ages 11–18)	11.3%	8.2%	10.7%	12.4%	13.9%
Wave II (ages 11–18)	13.9%	11.2%	12.7%	14.4%	17.3%
Wave III (ages 18–26)	24.5%	21.8%	24.3%	23.1%	29.1%
Wave IV (ages 24–32)	38.0%	35.2%	35.0%	37.8%	43.9%
Wave V (ages 32–42)	43.4%	38.2%	43.3%	43.3%	48.8%
High waist circumference					
Wave IV (ages 24–32)	49.2%	47.4%	49.4%	46.8%	53.0%
Wave V (ages 33–43)	51.2%	47.8%	51.1%	48.7%	56.9%
Diabetes					
Wave IV (ages 24–32)	5.9%	5.5%	4.2%	5.3%	8.6%
Wave V (ages 33–43)	8.4%	9.6%	5.4%	6.9%	11.4%
Hypertension					
Wave IV (ages 24–32)	24.4%	22.9%	24.3%	23.7%	26.8%
Wave V (ages 33–43)	31.8%	28.7%	34.4%	29.1%	35.5%
Hyperlipidemia					
Wave IV (ages 24–32)	16.6%	17.6%	17.7%	15.5%	15.7%
Wave V (ages 33–43)	14.9%	14.2%	16.4%	14.4%	14.5%

^a^All means and percentages are calculated with weighted data to reflect the representative proportion in the target U.S. population

^b^Household income was transformed into a continuous measure that was a ratio of household income relative to the federal poverty level

In mixed-effects generalized linear models adjusting for potential confounders (Table 2), each additional hour of screen time per day was associated with a 0.06 kg/m^2^ (95% confidence interval [CI] 0.04–0.09) within-person increase in BMI. Each additional hour of screen time per day was associated with 1.17 (95% CI 1.09–1.26) higher odds of high waist circumference, 1.09 (95% CI 1.03–1.15) higher odds of obesity, and 1.17 (95% CI 1.07–1.28) higher odds of diabetes in adjusted models accounting for within-person effects. Screen time was significantly associated with higher odds of hypertension in unadjusted models (1.10, 95% CI 1.06–1.14), though no association was found with hypertension (1.03, 95% CI 0.98–1.08) or hyperlipidemia (1.01, 95% CI 0.95–1.08) when adjusted for confounders.

**Table 2:** Associations Between Screen Time and Cardiometabolic Disease Outcomes in the National Longitudinal Study of Adolescent to Adult Health (1994–2018)

Cardiometabolic disease outcomes	Screen time (exposure, hours per day)			
	Unadjusted		Adjusted^a^	
Continuous outcomes	Coefficient (95% CI)	*p*	Coefficient (95% CI)	*p*
Body mass index change^b^	−0.15 (−0.18 to −0.12)	< 0.001	0.06 (0.04–0.09)	< 0.001
Waist circumference^c^	1.35 (1.17–1.53)	< 0.001	0.65 (0.50–0.79)	< 0.001
Binary outcomes	Odds ratio (95% CI)	*p*	Odds ratio (95% CI)	*p*
Obesity^b^	1.07 (1.04–1.10)	< 0.001	1.09 (1.03–1.15)	0.002
High waist circumference^c^	1.14 (1.08–1.21)	< 0.001	1.17 (1.09–1.26)	< 0.001
Diabetes^c^	1.25 (1.15–1.36)	< 0.001	1.17 (1.07–1.28)	< 0.001
Hypertension^c^	1.10 (1.06–1.14)	< 0.001	1.03 (0.98–1.08)	0.204
Hyperlipidemia^c^	1.01 (0.95–1.07)	0.783	1.01 (0.95–1.08)	0.677

Screen time based on average of screen time measures until the respective wave of the outcome measure

^a^Adjusted for age, sex, race/ethnicity, household income, highest education, smoking, alcohol, and baseline BMI

^b^Based on five repeated measures from Wave I to Wave V. Outcome was change from baseline BMI (Wave I)

^c^Based on two repeated measures from Wave IV to Wave V

Sensitivity analyses excluding participants classified as obese at Wave I (ESM Appendix B) and examining objective versus self-reported outcomes (ESM Appendix C) had similar findings to the main analysis. Average screen time in Waves IV and V was not associated with change in blood pressure or hemoglobin A1c between Waves IV and V (ESM Appendix D).

## DISCUSSION

This study used national longitudinal cohort data of adolescents followed over a 24-year period to analyze the relationship between screen time and cardiometabolic disease. We found that higher screen time from adolescence to middle adulthood was associated with multiple measures of cardiovascular health, including higher BMI, high waist circumference, obesity, and diabetes. Across all five waves of the study, prior screen time exposure was associated with an increase in BMI. Higher average screen time exposure was also associated with diabetes, though no association was found between average screen time and hypertension and high cholesterol when adjusted for confounders. Overall, these findings indicate that higher screen time may be associated with worse cardiometabolic health outcomes in later life.

Our findings are consistent with previous studies which have demonstrated positive associations between screen time and BMI and diabetes. Though there are few longitudinal studies to date, our findings are consistent with results from a New Zealand–based study that found a positive association between screen time and weight^15^ and expands the findings to a larger, older cohort in the U.S. While the effect size for BMI in the current study was relatively small (0.06 kg/m^2^ for each hour of screen exposure), several hours per day of screen exposure could be clinically significant, especially given adolescents are now reporting almost 8 hours per day of screen time, mostly watching or streaming videos, movies, or television shows.^6^

Several mechanisms have been suggested to explain the association between screen time and BMI, primarily centered around the idea that screen time is a sedentary behavior. Sedentary behavior displaces physical activity and can lead to increased caloric consumption through avenues such as advertisements that promote high-calories foods (e.g., fried foods, processed meat, sugar-sweetened beverages). Mindless snacking while watching television or videos could be another contributor.^4^ These mechanisms may also explain the association found between screen time and diabetes in the current study. This finding is consistent with a 2011 meta-analysis which reported that television viewing was associated with an increased risk of type 2 diabetes, cardiovascular disease, and all-cause mortality.^11^ The four studies examining the association between television viewing and type 2 diabetes identified by the meta-analysis were all limited to adults;^11^ therefore, our study adds to the literature by starting in adolescence. In addition to the mechanisms discussed above, television viewing can attract adolescents to unhealthy behaviors such as smoking, an established independent risk factor for type 2 diabetes and cardiovascular disease.^11^

Our study did not find an association between screen time and cholesterol, while previous studies, including the aforementioned New Zealand study,^15^ have reported an increase in serum cholesterol with screen time. Additionally, few studies have reported an association between screen time and blood pressure, with the current study finding a weak association between the two variables in unadjusted models but not after confounder adjustment. The main theoretical mechanism linking television viewing to CVD risk is through sedentary time; however, some individuals could be standing or active while watching television and the television viewing measure does not account for other sedentary activities.^43^ Further, it is possible that potential associations between screen time and CVD risk outcomes (e.g., hypertension and hyperlipidemia) may become detectable as the cohort ages past the third decade of life given the expected increased incidence of hypertension and high cholesterol in later adulthood.

Several limitations and strengths of our study should be noted. We acknowledge the current study’s lack of diversity in screen time measures, which only included total hours per week of television and video viewing. Though streaming of videos, movies, and television shows on screens such as smartphones, tablets, or computers is increasingly common,^6,36^ the displacement of physical activity and exposure to unhealthy advertisements and product placement remains relevant with these modalities. It is important that future studies account for these contemporary measures of screen time (smartphones, tablets, and computers) in order to characterize screen usage most accurately. Further, screen time measures were based on self-reported data, which could be subject to recall and reporting bias. The use of BMI as a measure of cardiometabolic disease should also be noted. While BMI is often recommended for evaluating obesity in children and adolescents, it does not distinguish between fat mass and lean body mass.^44^ Thus, individuals with increased muscle mass (e.g., athletes), may have a high BMI without an associated increase in cardiometabolic disease risk.^45^ Finally, certain measurements of metabolic data such as blood pressure, hemoglobin A1c, and cholesterol were only taken at later waves (i.e., Waves IV and V), limiting analysis of association between screen time and cardiometabolic disease risk over time. There is also the potential for unmeasured confounders. Strengths of the study include the long follow-up period of 24 years as well as the large, nationally representative cohort from which the data was drawn.

Overall, our findings indicate that screen time use in adolescence may be associated with higher cardiometabolic disease risk in later life. Adolescence is a critical period during which lifelong habits can develop.^46^ Given the increasing trends of screen time use among adolescents,^6^ our findings have important policy and public health implications, particularly as they relate to the development of screen time guidelines and interventions targeted at youth. Screen time guidelines should consider the long-term implications and risk associated with excessive screen time as demonstrated in our study. Currently, the Department of Health and Human Services 2018 *Physical Activity Guidelines for Americans* do not have recommendations for adolescent sedentary behaviors including screen time given insufficient evidence.^47^ Community- and school-led efforts could include investing resources in recreational facilities to encourage physical activity, digital media literacy, and education programming in the curriculum. Community centers may serve as a central location to provide educational outreach to students and parents regarding ways they can help monitor and reduce their children’s recreational screen time. Future longitudinal studies should continue to expand the follow-up period past age 43 to look for any associations that may arise in later life.

## Supplementary information

ESM 1 (DOCX 22 kb)

## References

[1] Carson V, Hunter S, Kuzik N (2016). Systematic review of sedentary behaviour and health indicators in school-aged children and youth: an update. Appl Physiol Nutr Metab.

[2] Thorp AA, Owen N, Neuhaus M, Dunstan DW (2011). Sedentary Behaviors and Subsequent Health Outcomes in Adults: A Systematic Review of Longitudinal Studies, 1996–2011. Am J Prevent Med.

[3] Vella CA, Taylor K, Nelson MC (2020). Associations of leisure screen time with cardiometabolic biomarkers in college-aged adults. J Behav Med..

[4] Saunders TJ, Chaput JP, Tremblay MS (2014). Sedentary Behaviour as an Emerging Risk Factor for Cardiometabolic Diseases in Children and Youth. Canadian J Diabetes.

[5] **Krantz-Kent R.** Television, Capturing America’s Attention at Prime Time and Beyond.; 2018:11. Accessed October 7, 2022. https://www.bls.gov/opub/btn/volume-7/television-capturing-americas-attention.htm

[6] Nagata JM, Cortez CA, Cattle CJ (2022). Screen Time Use Among US Adolescents During the COVID-19 Pandemic: Findings From the Adolescent Brain Cognitive Development (ABCD) Study. JAMA Ped.

[7] **Nagata JM, Singh G, Sajjad OM, et al.** Social epidemiology of early adolescent problematic screen use in the United States. *Pediatr Res* 2022;92(5):1443-1449. 10.1038/s41390-022-02176-8 10.1038/s41390-022-02176-8 PMC9243697 35768491

[8] **Anderson M, Jiang J.** Teens, Social Media, and Technology 2018.; 2018. Accessed October 7, 2022. https://www.pewresearch.org/internet/2018/05/31/teens-social-media-technology-2018/

[9] Fang K, Mu M, Liu K, He Y (2019). Screen time and childhood overweight/obesity: A systematic review and meta-analysis. Child: Care, Health Dev.

[10] Sivanesan H, Vanderloo LM, Keown-Stoneman CDG, Parkin PC, Maguire JL, Birken CS (2020). The association between screen time and cardiometabolic risk in young children. Int J Behav Nutr Phys Act..

[11] Grøntved A, Hu FB (2011). Television Viewing and Risk of Type 2 Diabetes, Cardiovascular Disease, and All-Cause Mortality: A Meta-analysis. JAMA.

[12] Nightingale CM, Rudnicka AR, Donin AS (2017). Screen time is associated with adiposity and insulin resistance in children. Arch Dis Childhood.

[13] Nagata JM, Iyer P, Chu J (2021). Contemporary screen time modalities among children 9-10 years old and binge-eating disorder at one-year follow-up: A prospective cohort study. Int J Eat Disord..

[14] Nagata JM, Iyer P, Chu J (2021). Contemporary screen time usage among children 9-10-years-old is associated with higher body mass index percentile at 1-year follow-up: A prospective cohort study. Pediatr Obes..

[15] Hancox RJ, Milne BJ, Poulton R (2004). Association between child and adolescent television viewing and adult health: a longitudinal birth cohort study. Lancet.

[16] **National Center for Injury Prevention and Control**. 10 Leading Causes of Death, United States. Published 2020. Accessed July 2, 2022. https://wisqars.cdc.gov/cgi-bin/broker.exe

[17] **Harris KM**. 2013 The Add Health Study: Design and Accomplishments. Carolina Population Center, University of North Carolina at Chapel Hill.

[18] **Harris KM, Halpern CT, Biemer P, Liao D, Dean SC**. Add Health Wave V Documentation: Sampling and Mixed-Mode Survey Design.; 2019. http://www.cpc.unc.edu/projects/addhealth/documentation/guides/

[19] **Entzel P, Whitsel E, Richardson A, et al.** Add Health Wave IV Documentation: Cardiovascular and Anthropometric Measures.; 2009. 10.17615/C6HM11

[20] **Whitsel E, Tabor JW, Nguyen QC, et al.** Add Health Wave IV Documentation: Measures of Glucose Homeostasis.; 2012. 10.17615/C64D4P

[21] **Whitsel E, Cuthbertson CC, Tabor JW, et al.** Add Health Wave IV Documentation: Lipids.; 2013. 10.17615/C6836K

[22] Badon SE, Littman AJ, Chan KG, Williams MA, Enquobahrie DA (2017). Trajectories of maternal leisure time physical activity and sedentary behavior during adolescence to young adulthood and offspring birthweight. Ann Epidemiol..

[23] Carter JS, Dellucci T, Turek C, Mir S (2015). Predicting Depressive Symptoms and Weight from Adolescence to Adulthood: Stressors and the Role of Protective Factors. J Youth Adolesc..

[24] Lee PH (2014). Association between adolescents’ physical activity and sedentary behaviors with change in BMI and risk of type 2 diabetes. PLoS One..

[25] Lee PH (2016). Physical activity, sedentary behaviors, and Epstein-Barr virus antibodies in young adults. Physiol Behav..

[26] Otten JJ, Littenberg B, Harvey-Berino JR (2010). Relationship between self-report and an objective measure of television-viewing time in adults. Obesity (Silver Spring)..

[27] Pettee KK, Ham SA, Macera CA, Ainsworth BE (2009). The reliability of a survey question on television viewing and associations with health risk factors in US adults. Obesity (Silver Spring).

[28] Vereecken CA, Todd J, Roberts C, Mulvihill C, Maes L (2006). Television viewing behaviour and associations with food habits in different countries. Public Health Nutr..

[29] Barlow SE, the Expert Committee (2007). Expert committee recommendations regarding the prevention, assessment, and treatment of child and adolescent overweight and obesity: summary report. Pediatrics.

[30] CDC. Defining Adult Overweight and Obesity. Centers for Disease Control and Prevention. Published June 3, 2022. Accessed June 23, 2022. https://www.cdc.gov/obesity/basics/adult-defining.html

[31] Clinical Guidelines on the Identification, Evaluation, and Treatment of Overweight and Obesity in Adults--The Evidence Report. National Institutes of Health. Obes Res. 1998;6 Suppl 2:51S-209S. 9813653

[32] American Diabetes Association. Diagnosis and classification of diabetes mellitus. Diabetes Care. 2014;37 Suppl 1:81. 10.2337/dc14-S081 10.2337/dc14-S081 24357215

[33] Whelton PK, Carey RM, Aronow WS (2018). 2017 ACC/AHA/AAPA/ABC/ACPM/AGS/APhA/ ASH/ASPC/NMA/PCNA guideline for the prevention, detection, evaluation, and management of high blood pressure in adults a report of the American College of Cardiology/American Heart Association Task Force on Clinical practice guidelines. Hypertension.

[34] Gooding HC, Milliren C, Shay CM, Richmond TK, Field AE, Gillman MW (2016). Achieving cardiovascular health in young adulthood-which adolescent factors matter?. J Adolescent Health: Off Publ Soc Adol Med.

[35] Shay CM, Ning H, Allen NB (2012). Status of cardiovascular health in US adults: prevalence estimates from the National Health and Nutrition Examination Surveys (NHANES) 2003-2008. Circulation.

[36] Nagata JM, Ganson KT, Iyer P (2022). Sociodemographic Correlates of Contemporary Screen Time Use among 9- and 10-Year-Old Children. J Pediatr..

[37] **Hamad R, Penko J, Kazi DS, et al.** Association of low socioeconomic status with premature coronary heart disease in US adults. JAMA Cardiol 2020;5(8):899-908. 10.1001/jamacardio.2020.1458 10.1001/jamacardio.2020.1458 PMC7254448 32459344

[38] **Nagata JM, Ganson KT, Griffiths S, et al.** Prevalence and correlates of muscle-enhancing behaviors among adolescents and young adults in the United States. Int J Adolesc Med Health 2020;34(2):119-129. https://doi.org/10.1515/ijamh-2020-0001 10.1515/ijamh-2020-0001 PMC9972881 32549173

[39] Nagata JM, Palar K, Gooding HC, Garber AK, Bibbins-Domingo K, Weiser SD (2019). Food Insecurity and Chronic Disease in US Young Adults: Findings from the National Longitudinal Study of Adolescent to Adult Health. J Gen Int Med.

[40] Irvine DS, McGarity-Shipley E, Lee EY, Janssen I, Leatherdale ST (2022). Longitudinal Associations Between e-Cigarette Use, Cigarette Smoking, Physical Activity, and Recreational Screen Time in Canadian Adolescents. Nicotine Tob Res..

[41] Curtis BL, Lookatch SJ, Ramo DE, McKay JR, Feinn RS, Kranzler HR (2018). Meta-Analysis of the Association of Alcohol-Related Social Media Use with Alcohol Consumption and Alcohol-Related Problems in Adolescents and Young Adults. Alcohol Clin Exp Res..

[42] **Chen P, Harris KM**. Guidelines for Analyzing Add Health Data. Carolina Population Center; 2020. 10.17615/C6BW8W

[43] Fox M (2012). What is sedentarism?. J Acad Nutri Dietetics.

[44] Styne DM, Arslanian SA, Connor EL (2017). Pediatric Obesity-Assessment, Treatment, and Prevention: An Endocrine Society Clinical Practice Guideline. J Clin Endocrinol Metab..

[45] Colpitts BH, Bouchard DR, Keshavarz M, Boudreau J, Sénéchal M (2020). Does lean body mass equal health despite body mass index?. Scand J Med Sci Sports..

[46] Sawyer SM, Azzopardi PS, Wickremarathne D, Patton GC (2018). The age of adolescence. Lancet Child Adolesc Health.

[47] 2018 Physical Activity Guidelines Advisory Committee. 2018 Physical Activity Guidelines Advisory Committee Scientific Report. U.S. Department of Health and Human Services; 2018.

