# Supplementary material for: Screen Time from Adolescence to Adulthood and Cardiometabolic Disease: a Prospective Cohort Study
Source: J Gen Intern Med. 2023 Jan 10;38(8):1821–7. doi: 10.1007/s11606-022-07984-6 (PMC10272026; doi:10.1007/s11606-022-07984-6)
Supplement: Supplementary file 1 — (DOCX 22 kb) [file 11606_2022_7984_MOESM1_ESM.docx]

| Appendix A. Attrition in the National Longitudinal Study of Adolescent to Adult Health | |
| --- | --- |
| **Wave** | n |
| Wave I (1994-1995, ages 11-18) | 20,745 |
| Wave II (1996, ages 11-18) | 14,738 |
| Wave III (2001-2002, ages 18-26) | 15,197 |
| Wave IV (2008-2009, ages 24-32) | 15,701 |
| Wave V (2016-2018, ages 32-42) | 12,300 |

| Appendix B. Associations between screen time and cardiometabolic disease outcomes in the National Longitudinal Study of Adolescent to Adult Health (1994-2018), excluding participants with baseline obesity | | | | |
| --- | --- | --- | --- | --- |
|  | Screen time (exposure, hours per day) | | | |
| Cardiometabolic disease outcomes | Unadjusted |  | Adjusted^a^ |  |
|  | Coefficient (95% CI) | p | Coefficient (95% CI)^b^ | p |
| Body mass index change^b^ | -0.15 (-0.18 - -0.12) | < 0.001 | 0.05 (0.03 - 0.08) | < 0.001 |
| Waist circumference^c^ | 0.97 (0.80 - 1.13) | < 0.001 | 0.65 (0.49 - 0.80) | < 0.001 |
|  | Odds ratio (95% CI) | p | Odds ratio (95% CI)^b^ | p |
| Obesity^b^ | 1.04 (1.01 - 1.08) | 0.011 | 1.08 (1.03 - 1.14) | 0.002 |
| High waist circumference^c^ | 1.12 (1.05 - 1.18) | < 0.001 | 1.18 (1.10 - 1.27) | < 0.001 |
| Diabetes^c^ | 1.26 (1.15 - 1.38) | < 0.001 | 1.18 (1.06 - 1.31) | 0.003 |
| Hypertension^c^ | 1.09 (1.04 - 1.14) | < 0.001 | 1.03 (0.98 - 1.09) | 0.217 |
| Hyperlipidemia^c^ | 0.99 (0.93 - 1.05) | 0.786 | 1.00 (0.94 - 1.07) | 0.960 |
| Screen time based on average of screen time measures until the respective wave of the outcome measure. | | | | |
| ^a^Adjusted for age, sex, race/ethnicity, household income, highest education, smoking, alcohol, and baseline BMI. | | | | |
| ^b^Based on five repeated measures from Wave I to Wave V. Outcome was change from baseline BMI (Wave I). | | | | |
| ^c^Based on two repeated measures from Wave IV to Wave V. | | | | |

| Appendix C. Associations between screen time and cardiometabolic disease (objective and self-report) outcomes in the National Longitudinal Study of Adolescent to Adult Health (1994-2018) | | | | |
| --- | --- | --- | --- | --- |
|  | Screen time (exposure, hours per day) | | | |
| Cardiometabolic disease outcomes | Unadjusted |  | Adjusted^a^ |  |
|  | Odds ratio (95% CI) | p | Odds ratio (95% CI)^b^ | p |
| Diabetes |  |  |  |  |
| Objective (labs or medications) | 1.23 (1.14 - 1.34) | < 0.001 | 1.13 (1.03 - 1.23) | 0.008 |
| Self-report | 1.24 (1.13 - 1.35) | < 0.001 | 1.18 (1.06 - 1.31) | 0.004 |
| Hypertension |  |  |  |  |
| Objective (blood pressure measurements or medications) | 1.10 (1.05 - 1.15) | < 0.001 | 1.04 (0.99 - 1.09) | 0.092 |
| Hypertension (self-report) | 1.11 (1.06 - 1.16) | < 0.001 | 1.05 (0.99 - 1.11) | 0.107 |
| Hyperlipidemia |  |  |  |  |
| Objective (labs or medications) | 1.00 (0.94 - 1.06) | 0.932 | 1.02 (0.95 - 1.08) | 0.636 |
| Hyperlipidemia (self-report) | 1.02 (0.97 - 1.08) | 0.349 | 1.04 (0.98 - 1.09) | 0.193 |
| Screen time based on average of screen time measures until the respective wave of the outcome measure. Cardiometabolic disease outcomes based on two repeated measures from Wave IV to Wave V. | | | | |
| ^a^Adjusted for age, sex, race/ethnicity, household income, highest education, smoking, alcohol, and baseline BMI. | | | | |

| Appendix D. Associations between screen time and change in cardiometabolic disease outcomes in the National Longitudinal Study of Adolescent to Adult Health (2008-2018) | | | | |
| --- | --- | --- | --- | --- |
|  | Screen time (exposure, hours per day) | | | |
| Cardiometabolic disease change outcomes | Unadjusted |  | Adjusted |  |
|  | Coefficient (95% CI) | p | Coefficient (95% CI)^b^ | p |
| Systolic blood pressure change^a^ | -2.68 (-58.32 - 52.96) | 0.924 | -13.56 (-67.22 - 40.10) | 0.618 |
| Diastolic blood pressure change^b^ | -2.68 (-58.58 - 53.21) | 0.924 | -13.61 (-67.56 - 40.35) | 0.619 |
| Hemoglobin A1c change^c^ | -0.07 (-0.20 - 0.06) | 0.318 | -0.01 (-0.15 - 0.13) | 0.867 |
| Screen time based on average of screen time measures until the respective wave of the outcome measure. Cardiometabolic disease outcomes based on two repeated measures from Wave IV to Wave V. Total cholesterol was reported in deciles in Wave IV so we were unable to calculate change in total cholesterol. | | | | |
| ^a^Adjusted for age, sex, race/ethnicity, household income, highest education, smoking, alcohol, and systolic blood pressure (Wave IV). | | | | |
| ^b^Adjusted for age, sex, race/ethnicity, household income, highest education, smoking, alcohol, and diastolic blood pressure (Wave IV). | | | | |
| ^c^Adjusted for age, sex, race/ethnicity, household income, highest education, smoking, alcohol, and hemoglobin A1c (Wave IV). | | | | |
